# Editorial: Advanced Self-assembled Materials with Programmable Functions

**DOI:** 10.3389/fchem.2022.892461

**Published:** 2022-04-13

**Authors:** Huacheng Zhang, Isurika R. Fernando, Jie Han, Kim Truc Nguyen, Jingbo Louise Liu

**Affiliations:** ^1^ School of Chemical Engineering and Technology, Xi’an Jiaotong University, Xi’an, China; ^2^ Department of Chemistry, Faculty of Applied Sciences, University of Sri Jayewardenepura, Nugegoda, Sri Lanka; ^3^ Key Laboratory of Advanced Energy Materials Chemistry (Ministry of Education), College of Chemistry, Nankai University, Tianjin, China; ^4^ William G. Lowrie Department of Chemical and Biomolecular Engineering, The Ohio State University, Columbus, OH, United States; ^5^ Department of Chemistry, Texas A&M University-Kingsville, Kingsville, TX, United States; ^6^ Texas A&M Energy Institute, College Station, TX, United States

**Keywords:** self-assembly, advanced functional materials, multi-dimensional materials, programmable functions, smart materials, supramolecular interactions, stimuli responsiveness

The process of self-assembly―where building units of a system organize into an ordered and/or functional structure via the internal arrangement of molecules―has attracted researchers from a broad range of disciplines which varies from chemistry and material science to engineering and technology. Over the last two decades, the new knowledge generated through the concept of “self-assembly” based fundamental research has opened up the doors for potential applications in engineering (Cao et al., 2021). In particular, this novel and inspiring methodological strategy of fabricating functional materials via self-assembly provides more opportunities for acquiring and optimizing the desired morphological and physicochemical properties of a material through proper design and synthesis of molecular building blocks. Thus, amalgamating the chemistry of self-assembly along with materials science will lead to the efficient fabrication of innovative materials with programmable functions.

Scientists involved in “self-assembly” related research not only focus on extending a significant fundamental study, but also target on solving practical issues in applied engineering during the development of advanced materials (Wang et al., 2019). The current research topic entitled as “Advanced Self-assembled Materials with Programmable Functions” embraced related but diverse research disciplines and areas such as organic chemistry, inorganic chemistry, supramolecular chemistry polymer chemistry, coordination chemistry, colloid and surface chemistry, biomaterials, environmental science, nanotechnology, nano-science, as well as functional materials science.

In fact, supramolecular chemistry was originated from mimicking of nature, especially some particular biological phenomena and catalytic studies (Zhang et al., 2020). In this research topic, Dang et al. provided an interesting view on protein dimerization with molecular approaches, and discussed opportunities for supramolecular chemistry in depth. Furthermore, Zhu et al. recently carried out the research of G-motif construction and chirality in deoxyguanosine monophosphate nucleotide complexes, revealing that supramolecular crystals overcomes the inherent limitations of self-assembly. In addition, Liu et al. employed the man-made active catalysts with Pd(0) and phosphine-based bulky ligand to synthesize the thiophene-containing conjugated polymers. Supramolecular interactions utilized in these researches including π−π stacking, charge transfer interactions, and metal-ion coordination driven self-assembly render novel mechanisms and efficient strategies to create materials whose properties could not be obtained only by using conventional covalent bonds. Here, Kong et al. investigated diverse driving forces such as hydrogen bonding and covalent bonding with tannic acid as the natural cross-linker for catalyst-free silicone elastomers. Additionally, those non-covalent driving forces were further employed in the potential application to biomedicines, for example, Abbasi et al. utilized the strong capacity of RNA aptamer B40t77 in specifically binding gp-120, and reported the liquid crystal based binding assay for detecting HIV-1 surface glycoprotein. Interestingly, these supramolecular interactions could further pave the way for preparing other advanced supramolecular architectures, such as mechanically interlocked molecules.

In particular, during the study of supramolecular interactions, scientists like to use the term “host” and “guest” to classify different roles among those valuable species. In this research topic, Sun et al. utilized traditional macrocyclic molecules to fabricate interesting calix[4]crown-based 1,3,4-oxadiazole, and further explored the possible application to fluorescent chemosensor for Cu(II) ion detections. Meanwhile, Huang and Tan. recently investigated the host-guest interactions between metal-organic frameworks and air-sensitive complex at high temperature. In fact, the concept of host-guest chemistry was not limited only in “organic” and “polymeric” substances, and a lot of “inorganic” materials could also fall under the concept of host and guest species (Li et al., 2020). Here, Sun et al. introduced microwave-assisted solvothermal method for preparing MoO_2_ nanospheres, which played a role as host material for the detection of H_2_S guest in wide concentration range at low temperature. In addition, Zhang et al. found that the general industrial by-product fluid catalytic cracking catalyst could be recycled as raw material, such as host species, for solidification of heavy metals, and guest species, for prevention of groundwater pollution caused by inadequate disposal.

Moreover, self-assembly could be used to control morphology and dimensions of thus obtained materials from the one dimensional (1D), two dimensional (2D), and three dimensional (3D) nanoscale, as well as make those materials programmable and reversible based on internal/external stimuli and by selectively introducing diverse functional groups (Fernando et al., 2015). In this research topic, Zhou et al. found that building blocks, especially efficient nitrogen-donors, could be used for building multifunctional 3D supramolecular co-coordination polymeric materials, which exhibited interesting applications in CO_2_ adsorption, antibacterial activity, as well as selective sensing of Fe(III)/Cr(III) ions and 2,4,6-trinitrophenol. Further, the reversible modifying method and diverse functional groups could assist in achieving different geometries/topologies of materials, leading to the construction of multi-dimensional smart materials. Here, Shinmori et al. studied porphyrin photoabsorption and fluorescence variation by adsorptive loading on gold nanoparticles. In addition, Dong et al. prepared self-assembled particle systems, and further carried out experimental investigations on profile control in Fuyu oilfield. Finally, advanced self-assembled materials with programmable functions could balance morphologies and physiochemical properties, and have wide prospective applications in crystals and chirality, chemosensors, biosensors, absorption of pollutants in air and ground water as environmental protection materials, biomaterials, and optical materials.
